# Population assessment of tropical tuna based on their associative behavior around floating objects

**DOI:** 10.1038/srep36415

**Published:** 2016-11-03

**Authors:** M. Capello, J. L. Deneubourg, M. Robert, K. N. Holland, K. M. Schaefer, L. Dagorn

**Affiliations:** 1 IRD, UMR MARBEC (IRD, Ifremer, Univ. Montpellier, CNRS), Sète, France; 2 Unit of Social Ecology, Université Libre de Bruxelles (ULB), Belgium; 3 Laboratoire de Technologie et Biologie Halieutiques, Institut français de recherche pour l’exploitation de la mer (Ifremer), Lorient, France; 4 Hawaii Institute of Marine Biology, University of Hawaii at Manoa, United States of America.; 5 Inter-American Tropical Tuna Commission (IATTC), La Jolla, United States of America.

## Abstract

Estimating the abundance of pelagic fish species is a challenging task, due to their vast and remote habitat. Despite the development of satellite, archival and acoustic tagging techniques that allow the tracking of marine animals in their natural environments, these technologies have so far been underutilized in developing abundance estimations. We developed a new method for estimating the abundance of tropical tuna that employs these technologies and exploits the aggregative behavior of tuna around floating objects (FADs). We provided estimates of abundance indices based on a simulated set of tagged fish and studied the sensitivity of our method to different association dynamics, FAD numbers, population sizes and heterogeneities of the FAD-array. Taking the case study of yellowfin tuna (*Thunnus albacares*) acoustically-tagged in Hawaii, we implemented our approach on field data and derived for the first time the ratio between the associated and the total population. With more extensive and long-term monitoring of FAD-associated tunas and good estimates of the numbers of fish at FADs, our method could provide fisheries-independent estimates of populations of tropical tuna. The same approach can be applied to obtain population assessments for any marine and terrestrial species that display associative behavior and from which behavioral data have been acquired using acoustic, archival or satellite tags.

Estimating the abundance of animal populations is central to modern ecology and conservation, both for terrestrial and marine species. This field continues to grow, not only because of the introduction of new statistical approaches and tools, but also due to the parallel technological advances that underpin new ways to conduct animal censuses through remote detection and telemetry^1^,^2^,^3^,^4^,^5^,^6^,^7^. Many survey methods used in terrestrial population ecology are based on the so-called distance-sampling approaches, where individuals are counted (or their signs, like animal footprints, droppings or sounds) over random points, quadrants or line transects^1^,^8^,^9^,^10^,^11^. From these measurements, absolute or relative abundance indices are derived by integrating the measured density of organisms over a certain area. Analogous methods have been employed in marine population ecology. Line transects have been widely used for small pelagic fish species using active acoustic techniques^12^,^13^,^14^. Equivalently, line transects based on visual inspections are commonly employed in abundance and diversity assessments of benthic species^15^,^16^. Additionally, aerial surveys allowed estimating the abundance for those species that are visible from the sea surface, like the Atlantic bluefin tuna^17^,^18^,^19^,^20^, whales^21^ and dolphins^22^. However, for the majority of large pelagic fish species, which are sparsely and patchily distributed in very large three-dimensional habitats, these types of surveys are difficult to conduct. An alternative approach for abundance estimates is the use of mark-recapture experiments. In this approach, which is widely employed in both terrestrial and marine ecology, animals collected in a series of samples are tagged and then released back into the population, where the marked animals are assumed to mix uniformly with the unmarked population. The total population is then estimated according to the ratio of marked to unmarked individuals that are recaptured^9^. However, conventional tagging data alone are rarely used to estimate the abundance of large pelagic fish and are generally employed in integrated assessment models along with other fisheries-dependent datasets (i.e. catch data) to obtain abundance estimates (see e.g. ref. 23). Besides, conventional tagging data are mainly exploited to estimate mortality and movement rates^24^,^25^,^26^. It is noteworthy that these conventional tagging projects also provide key information on the biology of large pelagic fish, such as growth rates and migration patterns^27^,^28^,^29^,^30^,^31^. However, although they allowed unprecedented sampling of subpopulations of animals, these approaches suffer possible bias when applied to pelagic fish species. Among other sources of error, these methods are affected by the disparate ways in which the fish are recaptured and require dedicated approaches to account for the erroneous reported recapture locations^32^. Additionally, there also may be problems with the way the marked individuals are distributed within the population that is being estimated. Finally, specific to marine ecology are those methods based on fisheries data, relying on the concept of catch-per-unit-of-effort (CPUE) indices^33^,^34^. These approaches are based on the idea that knowing how much effort is put into catching and removing fish from the population can provide a relative index of abundance, with the assumption that the same amount of effort will always remove the same proportion of the population that is present. However, rapid technological shifts in fisheries (and concomitant changes in harvest efficiency) make it difficult to analyze the time series of historical data and require dedicated standardization methods to account for this variability^34^. These CPUE indices are used in integrated assessment models in combination with conventional tagging data^23^.

The recent introduction of animal remote tracking technologies through satellite, archival and acoustic tagging allows unprecedented opportunities for gaining knowledge of the spatial and behavioral ecology of different species in their natural habitats. Thanks to this technology, marine scientists can now gain more insights about movements and behavior of large pelagic fish (e.g. refs 35, 36, 37, 38). However, despite these technological developments, few methods based on the knowledge of animal behavior have been proposed for estimating their abundance^3^,^39^,^40^. Here we propose a way to integrate telemetered behavioral data (specifically, the association of tropical tuna with floating objects, see below) into a new method of obtaining indices of population abundance. We argue that it is possible to use components of this associative behavior (namely, the residence and absence times at different aggregation sites), to estimate the size of local populations from which the groups of associated animals are drawn. This method is independent of understanding the causative factors that underpin the associative behavior. Specifically, we considered the issue of estimating tropical tuna abundance, taking advantage of their associative behavior with floating objects. Considering their ecological and economic importance and that currently no method exists for obtaining direct, fisheries-independent estimates of their populations, the development of new approaches for evaluating the abundance of tropical tuna is crucial. Different species of tropical tunas, such as skipjack (*Katsuwonus pelamis*), yellowfin (*Thunnus albacares*) and bigeye (*Thunnus obesus*) tuna are known to associate with natural or man-made floating objects, usually called Fish Aggregating Devices (FADs) and fishers have been exploiting this associative behavior for years^41^. In recent decades, this natural phenomenon has been exploited by purse seine tuna fisheries, which deploy a large number of drifting FADs to increase their chances to locate and catch tropical tuna^42^. In the following, we demonstrate, through modeling and data analysis, that knowledge of tropical tuna behavior around FADs and quantification of individual residence times around floating objects can provide a new path for direct estimates of populations of tropical tuna.

## Methods

### Model definition

We considered a system of *N* fish individuals in an array of *p* FADs^43^,^44^,^45^. A fish can be in one of the two following states: it can either be associated with one of the FADs, or be unassociated, i.e., occupy a portion of the sea outside of the zone of influence of any FAD. Considering that the total number of fish *N* is a conserved quantity (assume no recruitment and mortality of fish and balanced exit/entry fluxes of fish within the area and timescale of interest), the fish population at time *t* is a constant that can be expressed as:


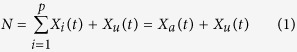


where *X*_*i*_(*t*) is the number of fish individuals associated to FAD *i* at time *t*, 
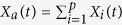
 is the total number of fish associated with all FADs and *X*_*u*_(*t*) is the number of unassociated fish. The time evolution of the number of associated fish is described through a system of *p* differential equations of the form^43^:


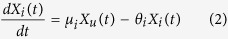


where *μ*_*i*_ denotes the probability for unassociated fish to associate with FAD *i* (with the index *i* = 1, … *p* running over all FADs) and *θ*_*i*_ denotes the probability for an associated fish to depart FAD *i* and become unassociated. Similarly, the time evolution of the number of unassociated fish reads:


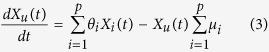


Considering equation (2) at equilibrium, the ratio between the number of associated fish at a given FAD *i* and the unassociated population can be expressed as:


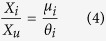


which implies 
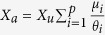
. Taking into account this relation and equation (1), we can write:


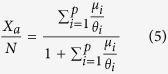


which provides the number of associated fish relative to the total fish population in terms of the parameters *μ*_*i*_ and *θ*_*i*_ that set the system’s dynamics (equation (2)).

Similarly, considering equation (5) for a specific FAD (denoted as FAD 1 in the following) leads:


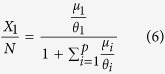


which provides the ratio between the population of fish associated with FAD 1 and the total population. Equation (6) implies that it is possible to relate the total population to the overall association dynamics and the population associated at one FAD only:


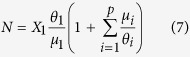


where *X*_1_ is the population associated at FAD 1.

### Derivation of abundance indices from continuous residence and absence times

Following the recent literature on FADs, the continuous bouts of time that individuals spend at the FADs or out of them are herein referred to as continuous residence times (CRT) and continuous absence times (CAT), respectively (see e.g. refs 45, 46, 47). By exploiting the methods of survival analysis, the model parameters in equation (2) can be inferred from the survival curves of CRTs and CATs^45^,^46^. The association dynamics defined in equation (2), where the probabilities *μ*_*i*_ and *θ*_*i*_ are two time-independent constants, implies a memoryless process with an exponential distribution of CRTs and CATs^45^. The survival curve of CRTs can be written as:


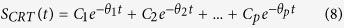


where *C*_*i*_ represent the proportion of CRTs recorded at FAD *i* and the arguments of the exponentials *θ*_*i*_ correspond to the probabilities to depart from FAD *i*. From the above relation, it is possible to infer the probabilities 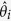 by fitting the survival curve of CRTs with a multiple exponential model. The coefficients *C*_*i*_ are related to the probability to associate with FAD *i* relative to the overall probability to associate with one of the *p* FADs and can be expressed in terms of the model parameters *μ*_*i*_ as follows:


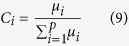


Similarly, the survival curve of CATs for a time-independent process follows:


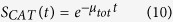


where 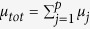 is the probability to associate with one of the *p* FADs. Combining equations (9) and (10) allows to infer the probability 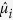 to reach FAD *i* as:


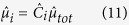


where 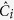 and 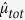 are estimated from the fits of the survival curves of CRTs and CATs with equations (8) and (10), respectively.

Equations (8) and (10) imply that the average CRTs and CATs can be related to 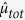 and 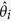 as follows:


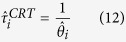


and


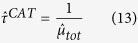


where 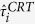 is the average continuous residence time recorded at FAD *i* and 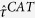 is the average continuous absence time spent off the FADs. Substituting equations (11, 12, 13) into equations (5) and (7) leads, respectively:


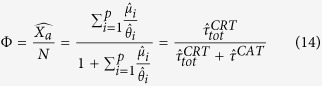


and


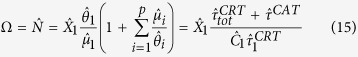


where 
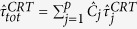
 is the average association time estimated over all FADs, 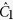 is the proportion of CRT recorded at FAD 1 and 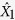 is the estimated population at FAD 1. Equation (14) provides the estimated ratio between the associated and total population form knowledge of the average residence and absence times only and the index Φ is thereafter referred to as *association index*. Similarly, the index Ω in equation (15) is thereafter referred to as the *abundance index*.

### Stochastic simulations: Algorithm description

The association dynamics described in equations (2, 3) was simulated by considering a system of *N* fish in an array of *p* FADs. Each fish individual was assigned to one of the *p* + 1 following states: either a fish was associated to one of the *p* FADs, or it was unassociated. At each time step *t* (with *t* = 1, . . . , *T*_*end*_), each of the unassociated fish *X*_*u*_(*t*) could move to FAD *i* (with *i* = 1, . . . , *p*) according to the probability *μ*_*i*_. Equivalently, each of the associated fish *X*_*i*_ at FAD *i* could depart from that FAD according to the probability *θ*_*i*_. The acceptance/rejection of the trial moves were implemented through comparison of *μ*_*i*_ and *θ*_*i*_ with a pseudo-random number *ξ* sampled from a uniform distribution in the interval (0, 1]. The trial move of departing a FAD *i* was accepted when *ξ* ≤ *θ*_*i*_. Equivalently, an unassociated individual moved to FAD *i* when 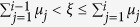. In the following, the choice in the model parameters ensured the positive-definiteness of the probabilities and the normalization conditions (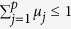 and *θ*_*i*_ ≤ 1). The initial position of all fish was assigned to the unassociated state and the system was let evolving in time following the above procedure, up to the end of the simulation at *t* = *T*_*end*_. For each time step we recorded the observables of interest: number fish in each of the *p* + 1 states and position of the fish individuals.

The simulations were run for 1000 replica. For each replica, the system’s properties were studied at equilibrium, when the average number of fish per FAD and outside of the FADs was constant in time. To this purpose, we excluded from the analysis a time lapse *T*_*start*_ located at the beginning of the simulation. At *T*_*start*_ a number of fish *N*_*T*_ (the so-called tagged fish) were sampled at FAD 1 (the FAD of tagging) among the *X*_1_ fish present at this FAD. The choice of following only a subset of individuals mimics electronic tagging experiments, where the number of tagged fish is generally much smaller than the total population present in a FAD array. For each of the *N*_*T*_ individuals we calculated the CRTs (CATs), as the continuous bouts of time spent at each FAD (outside of the FADs) without any interruption. For each tagged fish, each CRT was followed by a CAT (by construction) and the algorithm kept track of the series of CRTs and CATs sequentially recorded for each fish during the simulation. To this purpose, each tagged individual *i* was associated to a vector **v**^*i*^ = (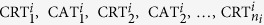), where 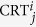 (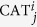) corresponds to the *j*^*th*^ CRT (CAT) recorded for fish *i* and 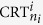 is the last CRT recorded during the simulation for individual *i* (notice that *n*_*i*_ can vary among individuals depending on the lengths of their CRTs/CATs). For each replica, the time-averaged number of associated fish 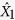 at FAD 1 (see equation (15)) was estimated from *T*_*start*_ up to the end of the simulation *T*_*end*_. In order to reproduce tagging experiments of different lengths, the average residence times and absence times (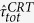, 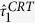 and 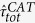), as well as the proportion of CRTs recorded at FAD 1 (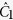) (see equations (14) and (15)) were estimated for variable numbers of CRTs and CATs. To this purpose, we considered a subset of CRTs/CATs 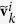 = (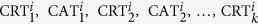) obtained from the individual vectors **v**^*i*^ defined above. Variable numbers of CRTs/CATs were obtained by pooling the vectors 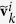 for increasing values of *k (k* being the same for all tagged fish and *k* < min(*n*_*i*_)). The association and abundance indices were then estimated for each replica following equations (14) and (15) and the average and standard deviation of each index calculated over the replica. The sensitivity of our results with respect to the tagging strategy was analyzed by plotting the association and abundance indices as a function of the total number of CRT considered. Also, the sensitivity of the indices relative to the inclusion of the first residence times recorded at the FAD of tagging (FAD 1) was analyzed, by including or excluding the first CRT recorded at FAD 1 (denoted below as CRT1). In the case where CRT1 was excluded, divergences in equation (15) due to the fact that no CRT were recorded at FAD 1 after the tagging, could lead to an undefined abundance index for some of the replica. This generally occurred when the total number of CRTs considered for each tagged individual was small relative to the probability to reach FAD 1. In order to avoid any bias, we considered values of the total number of CRTs where the abundance index was defined for all replica. For all case studies, the model was run for *T*_*start*_ = 1.0e4, *T*_*end*_ = 1.0e5 and *N*_*T*_ = 10 and a sensitivity analysis was conducted on the other model parameters, see paragraph below.

### Sensitivity analysis of the abundance indices

First, the model was run for a homogeneous system, where all the FADs had the same arrival and departure probabilities *μ*_*i*_ = *μ* and *θ*_*i*_ = *θ*. Here, the following case studies were considered: (i) variable association dynamics *μ*/*θ*, (ii) population sizes *N* and (iii) numbers of FADs *p*. For case study (i), the departure probability *θ* was fixed to a constant value and only the dependence on the association probabilities *μ* was studied, since the properties of the homogeneous system at equilibrium only depend on the ratio *μ*/*θ*^43^. Secondly, we studied a heterogeneous system with two different FAD classes, denoted as FAD-class A and B (with model parameters *μ*_*A*_, *θ*_*A*_ and *μ*_*B*_, *θ*_*B*_ respectively). Here, the FAD of tagging (FAD 1) was assigned to FAD-class A and the following case studies were considered: (iv) variable association probabilities *μ*_*B*_, (v) departure probabilities *θ*_*B*_ and (vi) numbers of FADs belonging to FAD-class A relative to class B, keeping constant all the other parameters. The model parameters considered for the homogeneous and heterogeneous case studies are reported in Tables 1 and 2, respectively.

### Experimental data analysis

**Figure 1:**
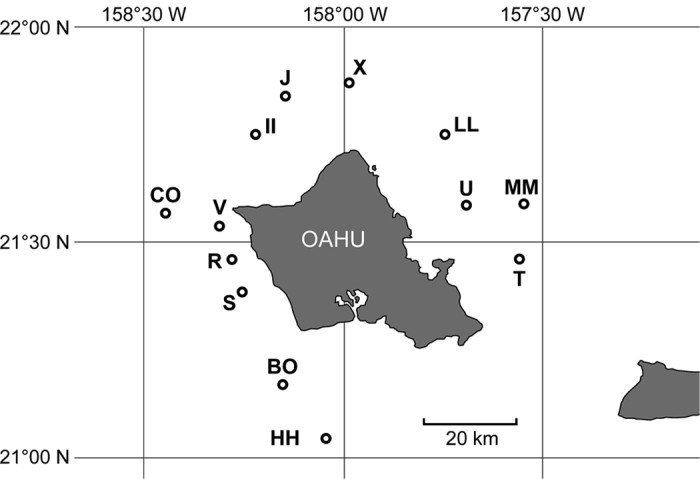
Map of the FAD array around the island of Oahu, Hawaii. Source: http://www.hawaii.edu/HIMB/FADS. Original Map was modified by P. Lopez (IRD) using Adobe Illustrator CS 2.

We considered a passive acoustic telemetry dataset collected from tagged yellowfin tuna monitored in an array of 13 instrumented FADs around the island of Oahu, Hawaii (USA), see Fig. 1. Details on the tagging procedure, tag specification and FAD array instrumentation can be found in refs 46 and 47 and in Appendix 1 of the Supplementary Material. We considered 28 yellowfin tuna with fork length larger than 50 cm tagged in 2003, see Supplementary Table S1. The choice of these individuals was based on previous studies that unveiled a homogeneous associative behavior for tagged individuals of this size class^48^. The choice of the time period was due to the fact that in 2003 the largest number of individuals of this size class was tagged and detected over a large portion of the FAD array. The association and absence times at/off the FADs were estimated using the definition of CRT and CAT, respectively, employed in ref. 46. Supplementary Table S2 resumes the CRTs recorded over the FAD array for the dataset.

The survival curves of CRTs and CATs were compared through the Cox proportional hazards regression model^49^, using the *survival* library^50^. Such comparison was performed for two purposes: i) assessing the classes of homogeneous FADs present in the array and ii) verifying that the equilibrium condition assumed in equation (4) was fulfilled. In order to assess the classes of homogeneous FADs, only the FADs with more than 10 CRTs recorded during the study period where analyzed individually. Reversely, for the FADs that were rarely visited, all the CRTs were pooled in a unique survival curve. The equilibrium condition was tested for each class of FADs by comparing survival curves of CRTs recorded over consecutive months that presented sufficient numbers of CRTs. The same test was run for CATs recorded over consecutive months. The hypothesis of time-independence in the association and departure probabilities (see equation (2)) was tested by fitting the survival curves of both CRTs and CATs with three models: exponential, double exponential and power law, using the *nls* function^50^. The first two models correspond to a memoryless, time-independent dynamics whereas the latter implies time-dependent probabilities to depart from and/or reach a FAD^45^,^46^, see Appendix 2 in the Supplementary material for more details. Goodness of fits was compared through the Akaike information criterion (AIC)^51^. When the AIC values of two models were close, the inspection of the standard errors of the model parameters and the principle of model parsimony drove the choice of the best fit. The probabilities of departing from FADs were inferred from the model fits of the survival curves of CRTs following equation (8) for each class of homogeneous FADs. The probabilities of reaching the FADs were estimated following equation (11). In this case, the sensitivity to the inclusion/exclusion of the first CRTs recorded after the tagging for each tagged individual (CRT1) was considered by including/excluding them in the proportion of CRTs (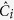 in equation (11)) recorded at the FAD-class of tagging (see Table S1). The association and abundance index were then estimated from equations (14) and (15) and the stochastic simulations described above were run using the inferred model parameters, in order to assess the sensitivity of the indices to the number of CRTs and the inclusion of CRT1.

### Ethic statement

The methods for handling and tagging yellowfin tuna were carried out in accordance with the relevant guidelines on animal experimentation and experimental surgery on fish. All experimental protocols were approved by the University of Hawaii Institutional Animal Care and Use Committee (IACUC).

## Results

### Results from stochastic simulations

#### Homogeneous system

**Figure 2:**
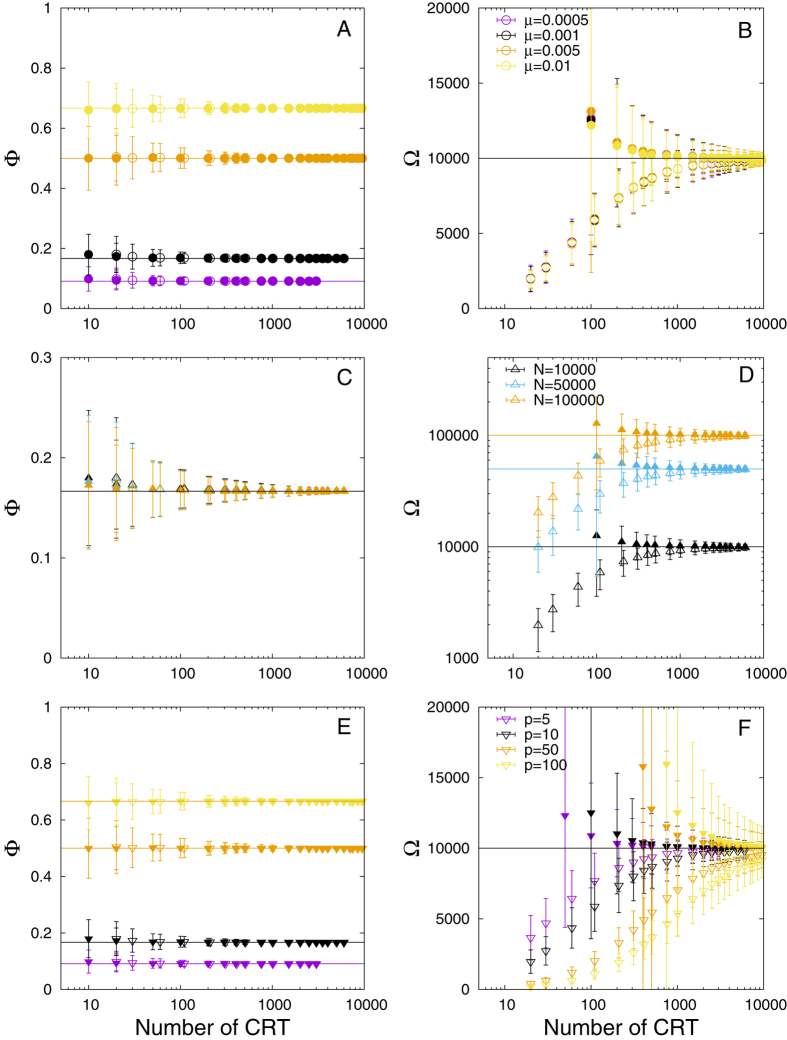
Homogeneous system. Association (left column) and abundance (right column) indices as a function of the total number of CRT. (**A,B**) Case study (i), with different probabilities to reach the FADs. (**C,D**) Case study (ii), with different population sizes. (**E,F**) Case study (iii) with different numbers of FADs. Empty/filled points correspond to the estimated indices with/without the first CRT recorded for each fish at the FAD of tagging (CRT1). The horizontal lines denote the asymptotic limits.

Globally, for a homogeneous system with equal probabilities of joining or departing from a FAD (Table 1), our model results indicate that the association index (equation (14)) is robust and shows little sensitivity to the tagging strategy and the model parameters (see Fig. 2A,C,E). On the other hand, the abundance index (equation (15)) was more sensitive to the number of CRTs (Fig. 2B,D,F). The inclusion of the first CRT recorded at the FAD of tagging (CRT1) in equation (15) led to an underestimated population for all case studies. When CRT1 was excluded from equation (15), the abundance index showed higher accuracies but larger variabilities. For all case studies, the asymptotic limit was reached for both indices for high numbers of CRTs. Case study (i) (Fig. 2A,B) demonstrated that both indices converged to the asymptotic values for any value of the association probability *μ* (Fig. 2A,B), the convergence being independent on the value of *μ*. Case study (ii) (Fig. 2C,D) demonstrate that both indices were not sensitive to the size of the population considered within the FAD array. Finally, changes in the total number of FADs (case study (iii)) resulted to be equivalent to varying *μ* for the association index (Fig. 2E). For the abundance index (Fig. 2F), including CRT1 in equation (15) produced higher losses in the accuracy for higher numbers of FADs. For example, the estimated population that showed high accuracies for 1000 CRTs and 10 FADs was lowered to nearly 50% of the true population in the presence of 100 FADs. This loss in accuracy was lower when excluding CRT1, but the variability of the abundance index increased when increasing the number of FADs.

#### Heterogeneous system

**Figure 3:**
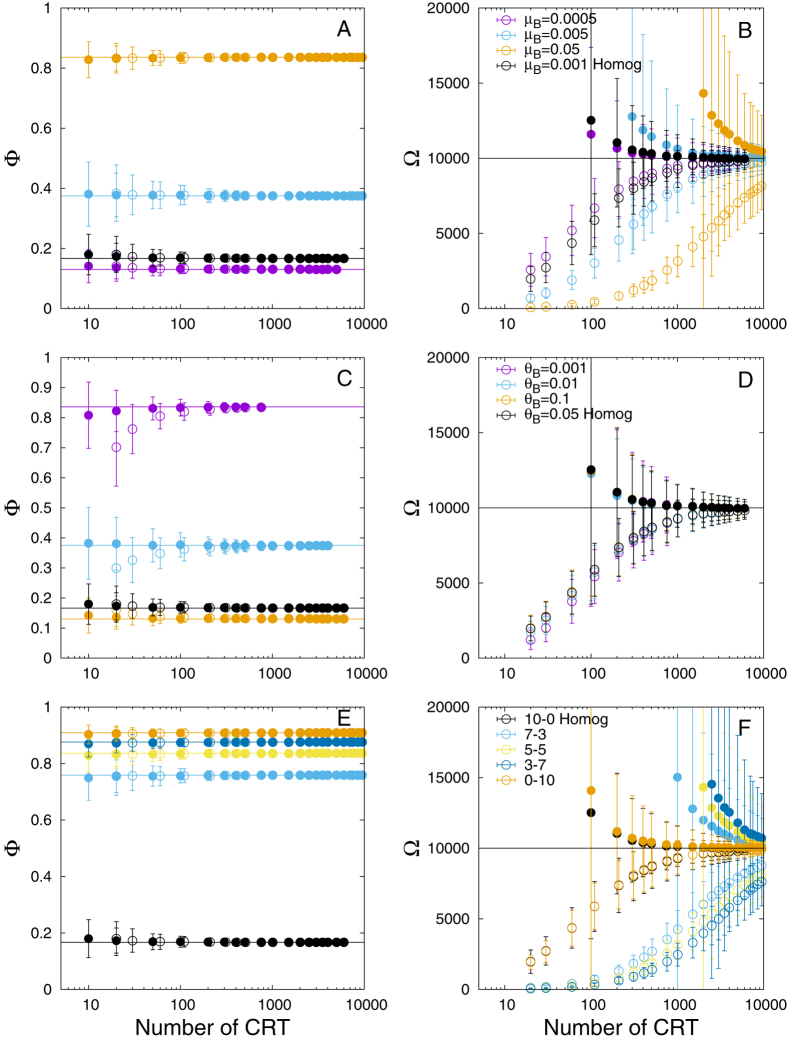
Heterogeneous system. Association (left column) and abundance (right column) indices as a function of the total number of CRT. (**A,B**) Case study (iv), with different probabilities to reach the FAD-class B. (**C,D**) Case study (ii), with different probabilities to depart from FAD-class B. (**E,F**) Case study (iii) with different proportions of FADs in FAD-class B relative to FAD-class A. Empty/filled points correspond to the estimated indices with/without the first CRT recorded for each fish at the FAD of tagging (CRT1). The black points denote the homogeneous system with parameters of FAD-class A. The horizontal lines denote the asymptotic limits.

All case studies converged to the asymptotic limit for large numbers of CRTs, see Fig. 3. Globally, the convergence of the association index showed little dependence on the properties of an heterogeneous array of FADs (Fig. 3A,D). The only exception was found for case study (v), where variable probabilities *θ*_*B*_ to depart from FAD-class B were considered (Fig. 3C). Here, when CRT1 was included in equation (14), smaller *θ*_*B*_ led to an underestimated association index. The abundance index showed an opposite trend. Higher dependencies on the model parameters and the tagging strategies were found for case studies (iv) and (vi) (Fig. 3B,F). When increasing the probability *μ*_*B*_ to reach FAD-class B (case study (iv) and Fig. 3B), the inclusion of CRT1 lead to an underestimated population. If CRT1 was excluded, the abundance index showed higher variabilities over the replica for larger values of *μ*_*B*_. Case study (vi) showed a similar trend to case study (iv), where larger proportions of FAD-class B induced lower accuracies and higher variabilities in the abundance index (Fig. 3F).

### Applications to tropical tuna in an array of FADs

The Cox proportional hazard model run over the survival curves of CRTs revealed two classes of FADs: FAD HH (denoted below as FAD-class 1) and FADs CO and V (FAD-class 2), see Supplementary Table S3. The CRTs recorded at the remaining FADs (FADs II, J, LL, R, T, X, MM, U, S and BO) were pooled and compared to these two classes of FADs. This comparison revealed that these FADs were not statistically different from FAD-class 2 (Supplementary Table S3). The Cox proportional hazard model run over the CRTs of FAD-classes 1 and 2 recorded at subsequent months demonstrated a temporal homogeneity for both FAD classes (Supplementary Table S4). The same result was obtained for CATs recorded at subsequent months, thus confirming that the equilibrium condition was attained (Supplementary Table S4). The comparison of goodness of fits between single exponential, double exponential and power law demonstrated that the survival curves of both CRTs recorded under FAD-class 1 and FAD-class 2 were exponentially distributed. FAD-class 1 was well described by a single exponential model and characterized by a mean CRT of 21.3 days. FAD-class 2 was best fitted by a double exponential model corresponding to two behavioral modes. Values of the AIC of exponential and power-law models were very close for FAD-class 1. Therefore, the exponential model was chosen for model parsimony, see Supplementary Table S5 and Appendix 4). The first behavioral mode represented 33% of the CRTs of FAD-class 2 with a mean duration of 0.07 days (1.7 hours), while the second behavioral mode represented 67% of the CRTs of FAD-class 2 with a mean duration of 3.7 days. Finally, the survival curves of CATs were best fitted by a single exponential model, see Supplementary Table S5, with a mean duration of 2.5 days for absence times. Table 3 resumes the optimal fitting parameters for both the CRTs and the CATs. Given the exponential survival curves of CRTs and CATs related to time-independent probabilities to depart from/reach the FADs and the fulfillment of the equilibrium condition, the model defined in equations (2, 3, 4, 5) was suitable to reproduce the observed association dynamics. In the presence of two FAD classes and a double exponential model for FAD-class 2 (Appendix 3 in the Supplementary Information), the association index can be written as:


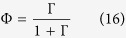


with:


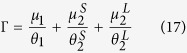


and in the limit 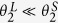:


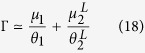


**Figure 4:**
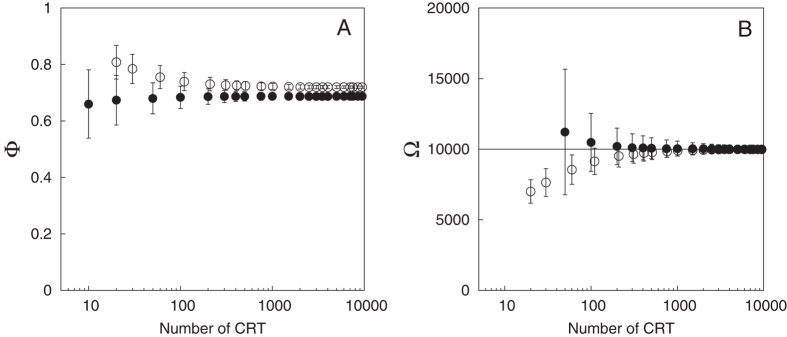
Field-based model. Association (**A**) and abundance (**B**) indices as a function of the total number of CRT. Empty/filled points correspond to the estimated indices with/without the first CRT recorded for each fish at the FAD of tagging (CRT1). The horizontal lines denote the asymptotic limits.

Estimates of the departure probabilities 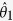, 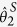 and 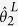 could directly be obtained from the fits of the survival curves of CRTs (Table 3). Moreover, the arrival probabilities 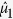, 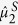 and 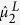 could be estimated from the fitted parameters in Table 3 through the following equations:


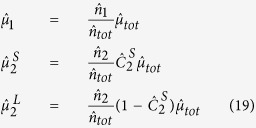


where 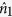 (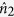) is the number of CRTs recorded at FAD-class 1 (FAD-class 2), 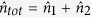 is the total number of CRTs, 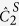 is the fraction of CRTs associated to short residence times for FAD-class 2 and 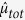 is the estimated probability to associate with one of the FADs of the array related to the survival curve of CAT, see equation (10). Application of the analytical formula (17) and (18) led to an association index of 0.721 (±0.087) and 0.720 (±0.086), respectively, which implies that the shortest timescales related to 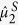 and 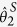 can be neglected and the asymptotic limit in equation (18) holds. Application of the analytical formula (18) with the exclusion of CRT1 in (19) led to an association index of 0.688 (±0.083), which is close to the previous values. Details for the derivation of the association index can be found in Appendix 5 of the Supplementary Material. The stochastic model was run with parameters obtained from the fitted values of Table 3 for 1000 replica, in the limit 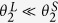, with and without taking into account CRT1 in the estimate of the model parameters (see Supplementary Table S6). The estimated association index showed little sensitivity relative to the number of CRTs recorded, see Fig. 4A. Secondly, the model could provide an estimate of the abundance index, in the hypothetical case where we could measure the population at FAD-class 1 (Fig. 4B). Table 4 reports the estimated association index and the estimated abundance index for a simulated population of 10 000 individuals. Relative errors of 1–3% and 5–10% were obtained for the association and abundance index, respectively, when the total number of CRT considered in the model was equal to 100 (i.e., of the same order of magnitude of the field experiment) thus providing indications that this approach can consistently be applied to realistic systems.

## Discussion

We propose a new way of deriving animal abundance (tuna in our case study) that relies on their associative behaviour around aggregating points and on the possibility of monitoring the dynamics of this behaviour through electronic tagging technologies. Our approach is based on knowing the amount of time spent by individuals at or away from aggregation points – in this case, using acoustic telemetry. These two measurable quantities can now be accessible through satellite, acoustic or archival tagging technologies in instances where it is possible to tag a subset of individuals of the species of interest and detect their characteristic associative behaviour. From knowledge of residence and absence times only, our approach provides the so-called association index, which informs on the fraction of the population that can be found at the aggregation points relative to the local population. Interestingly, when the total number of associated individuals at one of the aggregation points is known, our approach provides absolute abundance indices, linking directly the number of associated individuals to the local population present within the sampled region.

The sensitivity analysis conducted on the association index revealed that high accuracies can already be obtained for small number of CRTs (<100), both for homogeneous and heterogeneous FAD arrays. Reversely, the abundance index converged less rapidly and provided underestimated (overestimated) abundances at small number of CRTs when including (excluding) the first CRT recorded at the FAD of tagging (CRT1). Such bias is due to the error in the estimates of the proportion of residence times (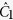 in equation (15)) spent at the FAD of tagging, which is overestimated (underestimated) when including (excluding) the first CRT recorded at the FAD of tagging. Despite the overall population is at equilibrium, the tagging of *N*_*T*_ fish is conducted at a single FAD (FAD1) so the tagged sub-population is out of equilibrium (being concentrated only at FAD1). The sensitivity analysis conducted herein on CRT1 allows to evaluate the effect of this out-of-equilibrium state of the tagged fish. Fishing and tagging operations are generally conducted on a subportion of the FAD array (for example, in our dataset of Hawaii the yellowfin tuna were tagged at only two FADs, CO and HH, see Table S1) and all data are generally included in the analyses to maximise the use of the information collected in the field. Ideally, one should wait for the system of tagged individuals to be at equilibrium. In practice, this equilibrium condition is difficult to assess because the number of tagged individuals in a FAD array is, unfortunately, relatively small and each of the tagged individuals spends a different amount of time in the FAD array. Here, we demonstrated that the abundance index may show a strong sensitivity to this tagging strategy and that the effect of including the first CRT should be considered in the estimated index.

Globally, for a homogeneous system, our analysis revealed that both indices showed little sensitivity in relation to the association dynamics and the total population. Reversely, the abundance index was highly sensitive to the number of FADs, with lower accuracies for higher numbers of FADs. Similarly to the trend of the index relative to the inclusion of CRT1, such bias can be explained by the increasing error in the estimates of (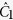) for larger numbers of FADs. This statement, however, should be considered with attention. The sensitivity analysis conducted here considers populations *at equilibrium* and with the same association dynamics. The approach does not intend to be predictive on how the association dynamics changes with respect to changes in the population size. It rather relies on the fact that, for any population size, if we can measure the association dynamics from field data, we can obtain accurate abundance estimates. Such equilibrium hypothesis relies on the fact that the behavioral timescales are much shorter than those related to the population dynamics. The same consideration should be taken for the other case studies considered in this paper, for example changes in the numbers of FADs (case study (iii)). The sensitivity analysis did not consider possible changes in the associative dynamics of tuna when the number of FADs is modified. The assumption here is that we can, in any case, measure the association dynamics for a given population size, number of FADs or FAD-array heterogeneities, and that the accuracies of the estimated association and abundance indices may depend on such variables.

The heterogeneous model allowed taking into account possible FAD-array heterogeneities, either induced by the characteristics of the FADs themselves, or by social interactions^43^. Our analysis was independent on the causative factors that induced such heterogeneities and the possible role of social interactions was indirectly taken into account by considering heterogeneous values of *μ*_*i*_ and *θ*_*i*_. For all case studies, the association index confirmed a high robustness also for a heterogeneous system. The only exception was case study (v) (heterogeneous probabilities of departing from the FADs), where, for *θ*_*B*_ ≪ *θ*_*A*_, the inclusion of CRT1 led to an underestimated association index. This happens because the proportion of CRTs associated to FAD-class B is underestimated relative to FAD-class A (where tagging occurs) and thus the association index is closer to the homogeneous case (*θ*_*B*_ = *θ*_*A*_) for small numbers of CRT. Reversely, the abundance index revealed a sensitivity to heterogeneous probabilities of joining the FADs, as well as on the relative number of FADs of each class. The best FAD for tagging fish and measuring the local biomass resulted to be the more attractive one (i.e., the one with higher *μ*_*i*_, thus with the best estimates of *C*_*i*_) and higher accuracies could be obtained when the FAD-class of tagging constitutes the majority of the array.

The application of this approach to field data was key for validating the main assumptions upon which the model is built: (i) time-independent dynamics and (ii) equilibrium condition satisfied. The hypothesis of time-independence in the probabilities of joining or departing from a FAD could be tested through the approaches of survival analysis. The survival curves of both CRTs and CATs could be best fitted by exponential models, which imply time-independent probabilities of joining or departing from a FAD. In some cases, power-law models also performed well. The distinction between multiple exponentials and power laws is not always straightforward, particularly for reduced number of points^52^. We therefore adopted the principle of parsimony and considered the simplest model that fitted well the experimental curves, thus choosing the exponentials models that best fitted the data. The equilibrium hypothesis found support from the comparison of survival curves of CRTs and CATs obtained at subsequent months, which demonstrate that the associative behavior of tuna did not change in time. Unfortunately, we did not dispose of measurements of the number of associated tuna, nor of its time evolution, to the purpose of assessing that equilibrium was attained at the level of the aggregation. Validating the equilibrium condition on the associated biomass (for example, through the echosounder buoys^53^) will undoubtedly be a key component of future studies of this type. Also, we could not verify the stationarity of the overall population. The fact that the tagged fish left the FAD array during the tagging experiment indicates that the possibility that yellowfin tuna leaves the FAD array certainly exists. However, verifying that other yellowfin tuna reached the FAD array in the meantime was not possible. Another important outcome of the method application to a realistic dataset was the assessment of the sensitivity of the association and abundance indices for an experimentally-observed association dynamics. To this purpose, the model parameters were derived from the fits of the survival curves of CRTs and CATs. In this respect, our results are promising and open the route to future applications of this method on tropical tuna and species showing the same associative behavior. We could demonstrate that for values of the model parameters inferred from field data, the accuracy of the association index is very high, already for a reduced numbers of residence times. Collecting field data of this kind is challenging: the number of fish that can be tagged is bounded by the high costs of the electronic tags and the issues of acoustic collisions that occur when too many individuals are tagged^45^,^54^. Our study demonstrates that such limits do not constitute a major issue for population assessments of tropical tuna. With the theoretical model we have demonstrated the possible bias induced by CRT1. In the analysis of experimental data, this translates in a biased estimate of *μ*_*i*_, the probability to reach the FADs where tagging operations have been conducted (equation 11). For this reason, we considered two possible values of *μ*_*i*_, one estimated with the inclusion of CRT1 and the other without CRT1. Reversely, all CRTs were included in the survival analysis of CRTs, assuming that there the effect of tagging on the residence times was negligible. This choice was dictated by the fact that we would reduce too much our database to have good survival curves when excluding CRT1. Also, in survival analysis, it is well known that left censoring does not introduce a bias, since the starting point (the beginning of CRT1) is defined by an event (fish tagging) which can be considered randomized relative to the time the tagged fish have already spent at the FAD and thus does not affect the properties of the survival curve.

Our model provides for the first time an estimate of the relative abundance of tropical tuna that can be considered to be within the general vicinity of an array of FADs. The estimated association index indicates high values for the ratio between the number of associated and total individuals around Oahu (Hawaii), of the order of 70%. One possible explanation for this high value could be that the individuals captured and tagged at the FADs are drawn from a subpopulation with a high “associative character”^47^. This possibility could be addressed by new tagging experiments where individuals would be tagged both at the aggregative points (FADs in the case of tuna) and away from them. Furthermore, based on the outcomes of the sensitivity analysis conducted theoretically (Fig. 2C,D), where the accuracy of the association and abundance indices was demonstrated to be independent on the size of the population, we could also test the robustness of the abundance index for realistic association dynamics. Assuming a population of 10 000 individuals and knowledge of the aggregated population at one FAD, we could provide an order of magnitude of the accuracy of our method. Remarkably, we found relative errors on the total population estimates ranging between of 3–10% and relative standard deviations of the same order of magnitude already for 100 CRTs, which indicate the applicability of this approach for abundance estimates of yellowfin tuna in a realistic experimental setting. Combined with estimates of the actual number of yellowfin tuna at Oahu FADs our method could provide an estimate of the local population around Oahu. In this respect, it is important to stress that the abundance index derived through this approach concerns only the fraction of the population of tropical tuna that can be found in association with FADs, i.e. skipjack tuna and small yellowfin and bigeye tuna.

A great advantage of this model is that it is simple and contains very few parameters: residence times at aggregation sites, residence times off aggregation sites and the population at one of the aggregating points are all that is required. In particular, equation (5) provides estimate of the relative abundance of associated individuals for a generalized aggregative system, independent of the mechanism that leads to the aggregation. Within our approach the effects of social interactions and the spatial heterogeneity induced by the environment can be naturally taken into account. The only assumption here is that the probability of joining or leaving the aggregation points is constant in time and that the population is stationary during the period of observation. For these reasons, this method is applicable to different species and case studies, not only to the one presented here. Each time animals show an associative behaviour, whatever the reason, it is possible to derive the relative abundance of associated animals for that region. This opens the possibility of using natural sites but also artificial sites that are designed specifically to aggregate animals and derive indices of abundance. This simplicity also implies that there is room for improvement, for example considering a spatialized model where the connectivity between FADs depends on their locations in the array.

Within the population-ecology literature, mark-recapture methods are generally employed for species that gather at specific locations, whereas distance-sampling methods are considered more effective for dispersed populations^11^. Our method can be considered as a combination of these two approaches. On one side, it is based on a subset of tagged individuals and exploits the aggregation of animals at particular points in space, increasing the detectability of those species that are otherwise dispersed over large areas. On the other side, it relies on detecting the presence/absence of these animals at the aggregating points. Its novelty and strength rely on the additional information obtained from the measurement of association and absence times. This information allows integrating the association dynamics within the sampling theory, thus accounting for the probability of observing (or not) the animal at the sampled aggregative points due to their behaviour.

More generally, our approach opens the route towards fisheries-independent abundance estimates of tropical tuna over wider regions, a crucial issue in fisheries management since no method currently exists for obtaining direct estimates of their populations. Using current generation archival tags, with light sensors, it is now possible to reconstruct the most probable tracks of individual fish and determine, for species exhibiting distinct diel vertical patterns depending on their type of association (e.g. bigeye tuna^55^), their associative history over several months and years. Equivalently, the long lifetime of acoustic tags now allows covering long periods of time. Furthermore, fishers currently use Global Positioning Systems (GPS) buoys with echosounders to obtain information on the presence and abundance of associated tuna (with no information on the species) around drifting FADs^56^. Access of these data would provide scientists with unprecedented large datasets regarding the amount of tuna at floating objects, which, when combined with tagging data, would allow first direct estimates of tuna populations^57^. The geographical extent of this population could be estimated by assessing the range of movements of fish caught and released at FADs in the region (as is currently occurring around anchored FADs in Hawaii using satellite telemetry techniques - Holland, unpublished data). Applying our approach would complement the current estimates obtained through more classical fisheries-dependent methods. In the hypothetical case where the number of fish at FADs remained high even if the wider population declined drastically, our method could really be innovative with respect to abundance estimates based on catch data. With our approach, we can estimate the proportion of associated population relative to the total population. Therefore, our abundance and association indices would be complementary to the fisheries-dependent abundance indices, and alert in the presence of “apparent” constant catches but an increasing ratio of associated population. Finally, our approach would allow the estimation of populations of other species that associate with floating objects, which represent the bycatch of tropical tuna purse seine fisheries. Populations of these species are rarely assessed due to lack of methods and data. Tagging these species would allow estimating their relative abundance and better evaluate the impacts of FAD fishing on pelagic ecosystems^42^.

In the same way that oceanographers have historically deployed different kinds of instrumented probes to study the physical dynamics of the oceans, it is now time for fisheries scientists to adopt the same strategy. By deploying networks of instruments that can observe the biological components of the oceans, both through FAD instrumentation and fish tagging, fisheries biologists can significantly improve their knowledge of the pelagic ecosystem. This new technology could be used to feed appropriate models with fishery-independent data that are vital for improved resource management strategies.

## Additional Information

**How to cite this article**: Capello, M. *et al.* Population assessment of tropical tuna based on their associative behavior around floating objects. *Sci. Rep.* **6**, 36415; doi: 10.1038/srep36415 (2016).

**Publisher’s note**: Springer Nature remains neutral with regard to jurisdictional claims in published maps and institutional affiliations.

## Supplementary Material

Supplementary Information

## Figures and Tables

**Table 1:** Model parameters for a homogeneous system.

Parameter symbol - Name	Case study (i)	Case study (ii)	Case study (iii)
*N* - Total number of fish	1.0e4	**1.0e4, 5.0e4, 1.0e5**	1.0e4
*p* - Total number of FADs	10	10	**5, 10, 50, 100**
*μ* -Probability to reach the FAD	**5e-4, 1e-3, 5e-3, 1e-2**	1.0e-3	1.0e-3
*θ* -Probability to depart from the FAD	5.0e-2	5.0e-2	5.0e-2
*N*_*T*_ - Number of tagged fish	10	10	10
*T*_*start*_ - Time of tagging	1.0e4	1.0e4	1.0e4
*T*_*end*_ - End Time of simulation	1.0e5	1.0e5	1.0e5

The cells in bold represent the model parameters that are varied in the sensitivity analysis. Case study (i) considers variations of the probability to reach the FADs (*μ*). Case study (ii) considers variable population sizes (*N*). Case study (iii) concerns variable numbers of FADs (*p*).

**Table 2:** Model parameters for a heterogeneous system.

Parameter symbol - Name	Case study (iv)	Case study (v)	Case study (vi)
*N* - Total number of fish	1.0e4	1.0e4	1.0e4
*p*_*A*_ - Total number of FAD-class A	5	5	**10, 3, 7, 0**
*p*_*B*_ - Total number of FAD-class B	5	5	**0, 7, 3, 10**
*μ*_*A*_ -Probability to reach FAD-class A	1.0e-3	1.0e-3	1.0e-3
*μ*_*B*_ -Probability to reach FAD-class B	**5.0e-4, 5.0e-3, 1.0e-3, 5.0e-2**	1.0e-3	5.0e-2
*θ*_*A*_ -Probability to depart from FAD class-A	5.0e-2	5.0e-2	5.0e-2
*θ*_*B*_ -Probability to depart from FAD class-B	5.0e-2	**1.0e-3, 1.0e-2, 5.0e-2, 1.0e-1**	5.0e-2
*N*_*T*_ - Number of tagged fish	10	10	10
*T*_*start*_ - Time of tagging	1.0e4	1.0e4	1.0e4
*T*_*end*_ - End Time of simulation	1.0e5	1.0e5	1.0e5

The cells in bold represent the model parameters that are varied in the sensitivity analysis. Case study (iv) concerns variable probabilities to reach FAD-class B (*μ*_*B*_). Case study (v) considers variable probabilities to depart from FAD-class B (*θ*_*B*_). Case study (vi) concerns variable numbers of FADs in class B relative to FAD-class A (*p*_*B*_).

**Table 3:** Optimized fitting parameters for the survival curves of CRTs and CAT obtained from the experimental data.

Data	n	Parameter	Estimate ± SD
CRT-Class 1	19	*θ*_1_	0.047 ± 0.002
CRT-Class 2	70	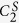	0.33 ± 0.01
		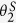	14.4 ± 1.8
		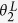	0.27 ± 0.01
CAT	61	*μtot*	0.396 ± 0.008

Columns (from left to right) indicate the data type (CRT/CAT and FAD class), the number of points for each survival curve, the estimated parameters and their associated standard deviation for the best fitting models, i.e., a single exponential (*S*_*CRT*_(*t*) = exp(−*θ*_1_*t*) for CRT and *S*_*CAT*_(*t*) = exp(−*μ*_*tot*_*t*) for CAT) and double exponential models (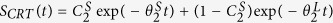 for CRT. Class 1 corresponds to CRTs recorded under FAD HH, and Class 2 under the other FADs of the array (CO, V, II, J, LL, T, X, MM, U, BO, S).

**Table 4:** Estimated association and abundance indices (average ± SD) obtained from the field-based model though equations (14, 15), for a total number of CRT equal to 100.

Index	Estimate	Estimate (*)	Rel. error	Rel. error (*)	Rel. SD	Rel. SD (*)
Association Ratio	0.74 ± 0.03	0.68 ± 0.04	3%	0.7%	4%	6%
Abundance index	9060 ± 1000	10500 ± 2000	9.4%	5%	10%	20%

Columns with (*) refer to estimates obtained when excluding the first CRT (CRT1) recorded at the FAD of tagging. Relative errors refer to the asymptotic values and relative SD are obtained by dividing the SD by the asymptotic value.
