# Supplementary material for: Population assessment of tropical tuna based on their associative behavior around floating objects
Source: Sci Rep. 2016 Nov 3;6:36415. doi: 10.1038/srep36415 (PMC5093414; doi:10.1038/srep36415)
Supplement: Supplementary Information [file srep36415-s1.pdf]

Population assessment of tropical tuna based on their associative behavior around floating objects

M. Capello, J-L. Deneubourg, M. Robert, K. Holland, K. Schaefer and L. Dagorn

Supplementary Material

Appendix 1 Details on the field data collection

The 13 FADs in Oahu are located between 21°E02′ – 21°E52′N latitude and 157° E33′–158°E27′ W longitude and are anchored in depths ranging between 500 and 2500 m. The distance between adjacent FADs ranges from 7.3 to 31.1 km. Each FAD was equipped with VEMCO VR2 acoustic receivers (www.vemco.com) before the start of the acoustic tagging campaigns. Tuna were captured within 500 m of FADs using surface trolling lures or baited lines with circle hooks. Coded VEMCO V16 tags (69 kHz, V16-4H-R256, 5–30 s delay, rated battery life 344 days) were inserted in the peritoneal cavity of healthy individuals. The tagging operations that concerned the 28 yellowfin tuna considered in this study took place between February and May 2003, see Table S1. The instrumented FAD array was operational during the entire release period and continued to be maintained and monitored until March 2005.

Appendix 2 Details on the models employed to fit the survival curves

The survival curves of residence and absence times were fitted using the three following equations:[46]

$$S(t) = e^{-k_1 t}, \tag{S1}$$

$$S(t) = f_1 e^{-k_1 t} + (1 - f_1)e{-}k_2 t \tag{S2}$$

and

$$S(t) = \beta^{\alpha}/(\beta + t)^{\alpha} \tag{S3}$$

The single exponential model in equation ((S1)) corresponds to a time-independent, memoryless process characterized by a single timescale $1/k_1$, where k_1 denotes the constant probability for a failure event to occur. The double exponential model in equation ((S2)) characterizes a time-independent, memoryless process associated to two timescales $1/k_1$ and $1/k_2$, with two constant probabilities k_1 and k_2. Here, f_1 represents the proportion of events associated to the timescale $1/k_1$. Finale the power-law model in equation ((S3)) describes a time-dependent dynamics, with α being the power coefficient and β being the minimal time for the first failure event to occur. In such model the probability for a failure event to occur decays with time as $\alpha/(\beta + t)$.

Appendix 3 Double exponential model for continuous residence times

Consider a fish population of size N, where the associated fish shows two possible behavioral modes relative to the time spent at the FADs, S and L (short and long residence times). The total number of fish associated with FAD i can be expressed as:

$$X_i = S_i + L_i \tag{S4}$$

where L_i (S_i) represents the amount of fish in state L (S) at FAD i. The total population is:

$$N = \sum_i^p (S_i + L_i) + X_u \tag{S5}$$

where X_u represents the number of unassociated fish. If the probabilities to join or depart from a FAD are time-independent, the number of associated fish in each behavioral state at FAD i is described by the following association dynamics:

$$\begin{aligned}\frac{dS_i}{dt} &= \mu_i^S X_u - \theta_i^S S_i \\ \frac{dL_i}{dt} &= \mu_i^L X_u - \theta_i^L L_i\end{aligned} \tag{S6}$$

where μ_i^S, μ_i^L, θ_i^S, θ_i^L are time-independent constants corresponding to the probability to reach (μ_i^S, μ_i^L) or depart (θ_i^S, θ_i^L) from FAD i. Considering equation ((S6)) at equilibrium leads:

$$\begin{aligned}S_i &= \frac{\mu_i^S}{\theta_i^S} X_u \\ L_i &= \frac{\mu_i^L}{\theta_i^L} X_u\end{aligned} \tag{S7}$$

From the above equations, the total number of associated fish can be expressed as:

$$X_a = \sum_i^p (S_i + L_i) = \sum_i^p \left(\frac{\mu_i^S}{\theta_i^S} + \frac{\mu_i^L}{\theta_i^L}\right) X_u \tag{S8}$$

and the ratio between the associated and total number of fish is given by:

$$\frac{X_a}{N} = \frac{\sum_i^p \left(\frac{\mu_i^S}{\theta_i^S} + \frac{\mu_i^L}{\theta_i^L}\right)}{1 + \sum_i^p \left(\frac{\mu_i^S}{\theta_i^S} + \frac{\mu_i^L}{\theta_i^L}\right)} \tag{S9}$$

The parameters on the r.h.s. of equation (S9) can be inferred from the survival curves of CRTs and CATs. In the presence of two behavioral states, the CRTs recorded at FAD i follow a double exponential model:

$$S_{CRT} = C_i^S e^{-\theta_i^S t} + (1 - C_i^S) e^{-\theta_i^L t} \tag{S10}$$

Where C_i^S represents the proportion of residence times for the behavioral state S and θ_i^S and θ_i^L are the two probabilities to depart from FAD i for a fish in behavioral state S and L, respectively. Reversely, the survival curves of CATs follow a single exponential model of the form:

$$S_{CAT} = e^{-\sum_i^p (\mu_i^S + \mu_i^L) t} = e^{-\mu_{tot} t} \tag{S11}$$

with $\mu_{tot} = \sum_i^p (\mu_i^S + \mu_i^L)$. From equation (S10), the values of θ_i^S and θ_i^L can directly be inferred from the fit of the survival curves of CRTs. Moreover, the probabilities μ_i^S and μ_i^L to reach FAD i for each behavioral state can be expressed as:

$$\begin{aligned}\mu_i^S &= \frac{n_i}{n_{tot}} C_i^S \mu_{tot} \\ \mu_i^L &= \frac{n_i}{n_{tot}} (1 - C_i^S) \mu_{tot}\end{aligned} \tag{S12}$$

where n_i is the number of CRTs recorded at FAD i and $n_{tot} = \sum_i n_i$ and C_i^S and μ_{tot} can be inferred from equations (S10) and (S11), respectively. Finally, considering the limit $\theta_i^S \gg \theta_i^L$, equation (S9) can be reduced to a single exponential model of equation (5), where the only relevant timescales are related to the long residence times $\theta_i = \theta_i^L$ and $\mu_i = \mu_i^L$.

Appendix 4 Details on the fit of the survival curves of CRTs and CATs

Survival curves of CRTs for FAD-class 1

The lowest values of the AIC were found for the double exponential model, see Table S5. However, one of the exponents of the double exponential function (k_1) was not significantly different from zero (p-value=0.16). As a consequence, the double exponential model was rejected. The AIC of the single exponential and the power-law models were very close, but the standard errors of the power law model were very high. For model parsimony, we therefore considered the single exponential model as the best fitting function for FAD-class 1.

Survival curves of CRTs for FAD-class 2

The double-exponential model showed the lowest AIC, which was significantly lower than the other models (Table S5). For this reason, this model was considered in the rest of the analysis.

Survival curves of CATs

The AIC of the single and double exponential models were very close (Table S5). However, one of the exponents of the double exponential function (k_2) was not significantly different from zero (p-value=0.7). As a consequence, the double exponential model was rejected. Reversely, the power-law model could not be fitted to the data, despite multiple trials were conducted by considering different initial conditions.

Appendix 5 Details on the model application to Yellowfin tuna

From the results obtained from the survival analysis of CRTs and CATs (see Results section and Appendix 4), the estimate of the abundance ratio took into account:

- An heterogeneous FAD array at equilibrium, with two classes of FADs, the fist class (FAD-class 1, with one FAD only) being characterized by a single exponential model and the second class (FAD-class 2, with 12 FADs) by a double exponential model.
- A single timescale related to a single exponential model for CATs.

In this case, the ratio between the associated and total number of fish can be analytically derived by combining equation (5) for a single exponential model and (S13) for a double exponential model for CRTs, leading to:

$$\Phi = \frac{X_a}{N} = \frac{\Gamma}{1+\Gamma} \tag{S13}$$

with:

$$\Gamma = \frac{\mu_1}{\theta_1} + \frac{\mu_2^S}{\theta_2^S} + \frac{\mu_2^L}{\theta_2^L} \tag{S14}$$

where μ_1, μ_2^S and μ_2^L (θ_1, θ_2^S and θ_2^L) represent the probabilities to associate with (depart from) the FADs of Class 1 and Class 2, respectively, considering for the latter two behavioral modes (S and L), see Appendix 3. The departure probabilities θ_1, θ_2^S and θ_2^L could be estimated from the fits of the survival curves of CRTs (see equation (8) and ((S9))). Similarly, the arrival probabilities μ_1, μ_2^S and μ_2^L could be estimated from field data through the following equations:

$$\begin{aligned} \mu_1 &= \frac{n_1}{n_{tot}}\mu_{tot} \\ \mu_2^S &= \frac{n_2}{n_{tot}}C_2^S\mu_{tot} \\ \mu_2^L &= \frac{n_2}{n_{tot}}(1-C_2^S)\mu_{tot} \end{aligned} \tag{S15}$$

where n_1 (n_2) is the number of CRTs recorded at FAD-class 1 (2), $n_{tot} = n_1 + n_2$ is the total number of CRTs, C_2^S is the fraction of CRTs associated to short residence times for FAD-class 2 (equation (S8)) and μ_{tot} is the probability to associate with one of the FADs of the array related to the survival curves of CAT (equation (S10)). In the case where CRT1 was not considered, the number of fish tagged at the FAD of each class (Table S1) were subtracted from the estimated values of n_1, n_2 and n_{tot} and the probabilities of joining each FAD class recalculated from equation (S15). Table S6 resumes the values of n_1, n_2 and n_{tot} with and without considering CRT1. Similarly, the approximate formula for the abundance ratio in the limit $\theta_2^L \ll \theta_2^S$ could be written as:

$$\Gamma = \frac{\mu_1}{\theta_1} + \frac{\mu_2^L}{\theta_2^L} \tag{S16}$$

The model parameters used in the stochastic simulations to reproduce the observed association dynamics are shown in Table S7. The model parameters of FAD class A correspond to those of FAD-class 1 (FAD HH) in the field data, whereas FAD-class B denotes the other FADs. The probabilities to reach each of the FADs of FAD-class B were derived from equation (S15) by dividing the estimated values of μ_2^L by the number of FADs that constituted that class (12 FADs).

Supplementary Tables

FAD	Year	Month	Number of Fish	Size (cm)
CO	2003	Feb	19	70.5 ±7
HH	2003	Mar, Apr, May	5,3,1	73.8 ± 4

Table S1. Tagging strategy: FAD of tagging, year and month of tagging, number of yellowfin tuna tagged and size range (mean fork length ± SD).

	Feb	Mar	Apr	May	June	July
BO	–	–	–	–	–	–
CO	10	13	–	–	–	–
HH	–	6	4	5	4	–
II	–	1	–	–	–	–
J	–	–	2	5	1	–
LL	–	–	–	1	1	–
MM	–	–	–	–	2	2
R	–	2	–	–	–	–
S	–	–	–	–	–	–
T	–	–	–	–	1	–
U	–	–	–	–	4	6
V	–	8	3	3	–	–
X	–	1	–	4	–	–

Table S2. Number of CRTs recorded at each FAD of the array during each month of the study period. For CRTs covering multiple months, the reported month indicates the end of the CRT.

Reference FAD	n	FAD Compared	n	z	$Pr(>\|z\|)$
HH	19	CO	23	3.869	0.00011
		V	14	4.808	1.5e-06
CO	23	V	14	-1.995	0.046
(CO,V)	37	(II,J,LL,MM,R,T,U,X)	33	-1.118	0.264
HH	19	(II,J,LL,MM,R,T,U,X)	33	3.724	0.000196

Table S3. Analysis of homogeneity among FADs. Results of the Wald test of comparison obtained from the Cox proportional hazard model run on survival curves of CRTs recorded at different FADs.

Data type	Reference	n	Comparison	n	z	Pr(> $|z|$)
CRT	FAD-class 1- March/April	10	FAD-class 1-May/June	9	-1.745	0.081
	FAD-class 2 -Feb	10	FAD- class2-March	25	0.74	0.460
CAT	March	19	April	10	-2.177	0.03
			May	12	-0.093	0.9

Table S4. Analysis of stationarity. Results of the Wald test of comparison obtained from the Cox proportional hazard model run on survival curves of consecutive months for the CRT recorded at each class of FADs and for CATs.

Survival curve	Model	Parameter	Estimate (SE)	$Pr(>|t|)$	AIC
CRT FAD-class 1	Single exp.	k_1	0.047 (0.002)	$<$ 1.4e-14	-57
	Double exp.	f_1	0.12 (0.04)	0.006	-70
		k_1	0.57 (0.38)	0.16	
		k_2	0.0388 (0.003)	4.96e-11	
	Power law	α	2.53 (0.98)	0.0187	-61
		β	43 (20)	0.047	
CRT FAD-class 2	Single exp.	k_1	0.65 (0.05)	<2e-16	-92
	Double exp.	f_1	0.33 (0.01)	<2e-16	-261
		k_1	14.4 (1.8)	2.3e-11	
		k_2	0.27 (0.01)	<2e-16	
	Power law	α	0.48 (0.03)	<2e-16	-211
		β	0.20713 (0.03)	5.09e-09	
CATs	Single exp.	k_1	0.396 (0.008)	$<$ 2e-16	-207
	Double exp.	f_1	0.98 (0.02)	$<$ 2e-16	-205
		k_1	0.41 (0.02)	$<$ 2e-16	
		k_2	-0.013 (0.04)	0.733	
	Power law	α	-	-	-
		β	-	-	

Table S5. Results for the fits of the survival curves of CRTs and CATs. The form of the three curves follow Appendix 2.

FAD-class	n	n (*)
FAD-class 1	19	10
FAD class 2	70	51
TOTAL	89	61

Table S6. Number of CRTs recorded for each FAD class. Last column denoted with (*) corresponds to the number of CRTs recorded in the array without considering CRT1.

**Parameter symbol - Name**	Value	Value (*)
N - Total number of fish	1.0e4	1.0e4
N_T - Number of tagged fish	10	10
p_A - Total number of FADs in class A	1	1
p_B - Total number of FADs in class B	12	12
μ_A - Probability to reach FAD-class A	0.0850	0649
μ_B - Probability to reach FAD-class B	0.0174	0.0184
θ_A - Probability to depart from FAD-class A	0.047	0.047
θ_B - Probability to depart from FAD-class B	0.27	0.27
T_{start} - Time of tagging	1.0e4	1.0e4
T_{end} - End Time of simulation	1.0e5	1.0e5

Table S7. Model parameters for reproducing the experimental data. The shaded cells represent the model parameters that are obtained from the experimental data (Table 3). Last columns denoted with (*) correspond to model parameters with probabilities of joining the FADs obtained when excluding CRT1. Notice that the probability to reach a single FAD of FAD-class B is obtained by considering μ_2^L in equation (S15) and dividing by the total number of FADs of class B p_B. The values of the other parameters are the same as in the stochastic simulation.
